# Lymph node yield less than 12 is not a poor predictor of survival in locally advanced rectal cancer after laparoscopic TME following neoadjuvant chemoradiotherapy

**DOI:** 10.3389/fonc.2022.1080475

**Published:** 2022-12-08

**Authors:** Hong Yang, Jiadi Xing, Chenghai Zhang, Zhendan Yao, Xiuxiu Wu, Beihai Jiang, Ming Cui, Xiangqian Su

**Affiliations:** Key Laboratory of Carcinogenesis and Translational Research (Ministry of Education), Department of Gastrointestinal Surgery IV, Peking University Cancer Hospital & Institute, Beijing, China

**Keywords:** locally advanced rectal cancer, lymph node yield, neoadjuvant chemoradiotherapy, laparoscopic surgery, prognosis

## Abstract

**Purpose:**

Previous studies have confirmed that neoadjuvant chemoradiotherapy (nCRT) may reduce the number of lymph nodes retrieved in rectal cancer. However, it is still controversial whether it is necessary to harvest at least 12 lymph nodes for locally advanced rectal cancer (LARC) patients who underwent nCRT regardless of open or laparoscopic surgery. This study was designed to evaluate the relationship between lymph node yield (LNY) and survival in LARC patients who underwent laparoscopic TME following nCRT.

**Methods:**

Patients with LARC who underwent nCRT followed by laparoscopic TME were retrospectively analyzed. The relationship between LNY and survival of patients was evaluated, and the related factors affecting LNY were explored. To further eliminate the influence of imbalance of clinicopathological features on prognosis between groups, propensity score matching was conducted.

**Results:**

A total of 257 consecutive patients were included in our study. The median number of LNY was 10 (7 to 13) in the total cohort. There were 98 (38.1%) patients with 12 or more lymph nodes harvested (LNY ≥12 group), and 159 (61.9%) patients with fewer than 12 lymph nodes retrieved (LNY <12 group). There was nearly no significant difference between the two groups in clinicopathologic characteristics and surgical outcomes except that the age of LNY <12 group was older (P<0.001), and LNY <12 group tended to have more TRG 0 cases (P<0.060). However, after matching, when 87 pairs of patients obtained, the clinicopathological features were almost balanced between the two groups. After a median follow-up of 65 (54 to 75) months, the 5-year OS was 83.9% for the LNY ≥12 group and 83.6% for the LNY <12 group (P=0.893), the 5-year DFS was 78.8% and 73.4%, respectively (P=0.621). Multivariate analysis showed that only patient age, TRG score and ypN stage were independent factors affecting the number of LNY (all P<0.05). However, no association was found between LNY and laparoscopic surgery-related factors.

**Conclusions:**

For LARC patients who underwent nCRT followed by laparoscopic TME, the number of LNY less than 12 has not been proved to be an adverse predictor for long-term survival. There was no correlation between LNY and laparoscopic surgery-related factors.

## Introduction

Rectal cancer is one of the most common malignant disease worldwide. In the National Comprehensive Cancer Network’s (NCCN) clinical practice guidelines, neoadjuvant chemoradiotherapy (nCRT) followed by total mesorectal excision (TME) is recommended as the standard of care for locally advanced rectal cancer (LARC) (1). It has been confirmed that nCRT can improve the local control and overall survival in patients with LARC without increasing the risk of serious complications compared with TME with no prior treatment (2–5). The American Joint Committee on Cancer (AJCC) recommends that at least 12 lymph nodes be examined to ensure accurate tumor staging (6). However, nCRT is known to significantly reduce the number of lymph node yield (LNY) in surgical specimens (7–10). Although this finding has been interpreted by some investigators as indicating a good response to nCRT and therefore a predictor of positive outcome (11, 12), others have suggested that detection of fewer lymph nodes may lead to understaging and staging migration, affecting patient outcomes (13, 14).

In recent years, several multicentre studies, such as the MRC-CLASSIC, COLOR II, and COREAN trials, have demonstrated that laparoscopic resection of rectal cancer is superior to open surgery in short-term efficacy with no significant difference in the aspect of oncological results (15–17). Therefore, laparoscopic surgery in rectal cancer has been rapidly promoted worldwide in the past decade. Laparoscopic surgery has a better surgical field of view than open surgery, making it easier for surgeons to identify anatomical landmarks and enter into the correct anatomical plane. In theory, laparoscopic techniques may potentially lead to some changes in lymph node dissection.

In this study, we investigated the relationship between LNY and survival in LARC patients who underwent laparoscopic TME following nCRT. In addition, characteristics associated with LNY were analyzed to determine whether laparoscopic surgery-related factors have an impact on LNY.

## Patients and methods

### Study population

Patients with locally advanced mid-low rectal cancer (cT3/T4 or N+, and lower edge of the tumor within 10 cm from the anal verge) who underwent nCRT followed by laparoscopic TME at the Department of Gastrointestinal Surgery IV, Peking University Cancer Hospital from January 2010 to December 2018 were collected from a prospectively maintained database. The exclusion criteria were as follows: 1. Patients received open surgery; 2. Patients who did not receive nCRT; 3. Patients underwent palliative resection or emergency surgery; 4. Patients with simultaneous distant metastases; 4. The interval from the completion of radiation to surgery more than 16 weeks; 5. Concomitant with other tumors or a history of other tumors within 5 years. Preoperative clinical assessment included digital rectal examination, routine blood testing, serum carcinoembryonic antigen (CEA) and carbohydrate antigen 19-9 (CA19-9) levels, colonoscopy biopsy, computed tomography (CT) of the chest and abdomen, pelvic magnetic resonance imaging (MRI) and endorectal ultrasonography (EUS). According to the number of lymph node dissection confirmed by postoperative pathology, the patients were divided into lymph nodes ≥12 group and < 12 group. The clinical data and long-term survival were compared between the two groups. This study was approved by the Research Ethics Committee of Peking University Cancer Hospital & Institute.

### Perioperative treatment

All patients in this cohort underwent preoperative nCRT. The vast majority of patients received long-term radiotherapy (usually a total dose of 50.6 Gy divided into 22 daily fractions), while only a few patients received short-term radiotherapy (usually a total dose of 30 Gy divided into 10 daily fractions). The most common concurrent chemotherapy regimen was continuous oral capecitabine (825 mg/m^2^ twice daily) during radiotherapy, and a small number of patients received 2-3 cycles of oxaliplatin-containing regimen, including CAPEOX (intravenous oxaliplatin 130 mg/m^2^ on day 1 plus oral capecitabine 1000 mg/m^2^ twice daily on days 1-14 every 3 weeks) or mFOLFOX6 (intravenous oxaliplatin 85 mg/m^2^ plus leucovorin 400mg/m^2^, 5-fluorouracil 2400mg/m^2^ on day 1 every 2 weeks). All patients in this study completed nCRT. Laparoscopic TME surgery was usually performed within 6 to 10 weeks after the completion of long-term radiotherapy and within 3 to 4 weeks after the completion of short-term radiotherapy. If the patient had a successful postoperative recovery, 3-6 months of 5-fluorouracil-based adjuvant chemotherapy (CAPEOX, mFOLFOX6 or capecitabine only) was routinely recommended around 4 weeks after surgery.

### Surgery and pathology

All the operations were performed laparoscopically by an experienced surgical team according to the principles of TME. Sphincter-preserving or non-preserving surgery was primarily depended on the location and stage of tumor, together with the surgeon’s experience and intraoperative judgment. The central lymph nodes were dissected, regardless of high or low inferior mesenteric artery ligation. Sharp separation was performed along the surgical plane between the mesentery and parietal fascia, and the autonomic nerves were preserved. Low anterior resection (LAR) was the main surgical procedure for patients who were suitable for retention of anal function, or else patients would receive non-preserving surgery, including abdominoperineal resection (APR), extralevator abdominoperineal excision (ELAPE) and Hartmann’s procedure. For sphincter-preserving surgery, diverting ileostomy was performed in patients with high-risk anastomosis. Postoperative complications were graded using the Clavien-Dindo classification (18).

The pathological examinations were performed by two pathologists independently. The TME quality was graded using the criteria of Nagtegaal et al. (19) as complete, nearly complete, or incomplete. Tumor stage was assessed according to the AJCC TNM staging system (the eighth edition) (20). Preoperative chemoradiotherapy response was classified in accordance with tumor regression grade (TRG) score recommended by AJCC, defined as follows: TRG 0 (complete response), no residual cancer cell; TRG 1 (moderate response), single or a cluster of cancer cells; TRG 2 (minimal response), residual cancer outgrown by fibrosis and TRG 3 (poor response), nearly no cancer cells killed, extensive residual cancer (21). CRM≤ 1 mm was considered positive.

### Follow-up

All patients were followed up every 3 months during the first 2 years after surgery, every 6 months in the following 3 years, and once a year thereafter. Follow-up assessment included a physical examination, routine blood testing, serum CEA, CA19-9 and other necessary examinations. Chest radiography or CT, abdominopelvic CT were conducted every 6 months, and colonoscopy was carried out annually. Overall survival (OS) was defined as the time from the date of surgery to the date of death from any cause. Disease-free survival (DFS) was defined as the time from the date of surgery to any type of relapse.

### Statistical analysis

Categorical variables were presented as numbers (percentages) and compared using the chi-square test or Fisher exact test. Continuous variables were presented as median (Inter quartile range, IQR) and compared using the Mann-Whitney U test. To further eliminate the differences in clinicopathological features between the two groups (LNY ≥12 or LNY <12), patients were matched 1:1 by propensity score (nearest neighbor matching with logistic regression, caliper 0.01 without replacement) using the covariates sex, age, pathological T stage, N stage, TNM stage and TRG score. Survival curves were drawn by the Kaplan–Meier method and compared using log-rank test. To identify independent determinants of the number of lymph nodes retrieved, a multivariable linear regression analysis was performed, and to explore risk factors affecting tumor recurrence, logistic regression analysis was conducted. All variables with potential significance in the univariate analyses were included in the multivariate analyses (based on a P value <0.1). Statistical analyses were performed using SPSS software (version 27.0, IBM Corporation), and P values <0.05 were considered statistically significant.

## Results

### Patient characteristics

A total of 257 consecutive patients with locally advanced mid-low rectal cancer treated with nCRT followed by laparoscopic TME in our center from January 2010 to December 2018 were included in this study. The median follow-up was 65 (54 to 75) months. The clinical and pathological characteristics of the patients are presented in **Tables 1**, **2**. The surgical outcomes are shown in **Table 3**. Of the 257 patients, males and females accounted for 63.4% and 36.6%, respectively. The median age of the whole group was 59 (52 to 65) years. The median height of the tumor from the anal verge was 5 (3 to 7) cm. A total of 239 (93.0%) patients received long-term radiotherapy, while only 18 (7.0%) patients received short-term radiotherapy. Most of the patients (81.7%, 210 of 257) received concurrent oral administration of capecitabine during radiotherapy, while a small number of patients (18.3%, 47 of 257) received combined chemotherapy regimen including oxaliplatin. In all patients, 101 (39.3%) patients were operated on before 2016 and another 156 (60.7%) after that, while the vast majority (86.8%, 223 of 257) performed by senior surgeons and only a few (13.2%, 34 of 257) performed by junior surgeons. The proportions of different surgical procedures were 49.0% for LAR, 42.0% for ELAPE, 7.4% for APR and 1.6% for Hartmann’s procedure, respectively. Within 30 days after surgery, the complication rate was 11.3%, the reoperation rate was 1.9%, and the mortality rate was 0.4%. Pathological data revealed that 15.2% (39 of 257) of the patients achieved pathologic complete response (pCR or/ypT0N0M0). The median number of lymph nodes detected was 10 (7 to 13) in all patients. A total of 98 (38.1%) patients had 12 or more lymph nodes harvested (LNY ≥12 group), and 159 (61.9%) patients had fewer than 12 lymph nodes retrieved (LNY <12 group).

**Table 1:** Comparison of demographic characteristics between LNY ≥ 12 group and < 12 group.

Variables	All (n=257)	Before matching			After matching		
		LNY ≥ 12	LNY < 12	*P value*	LNY ≥ 12	LNY < 12	*P value*
		(n=98)	(n=159)		(n=87)	(n=87)
Sex, n (%)				0.196			0.634
Male	163 (63.4)	67 (68.4)	96 (60.4)		58 (66.7)	55 (63.2)	
Female	94 (36.6)	31 (31.6)	63 (39.6)		29 (33.3)	32 (36.8)	
Age, median (IQR), years	59 (52- 65)	55 (50- 63)	60 (54- 65)	0.001	56 (50-63)	57 (52-63)	0.575
ASA, n (%)				0.410			1.000
I	56 (21.8)	24 (24.5)	32 (20.1)		21 (24.1)	21 (24.1)	
II-III	201 (78.2)	74 (75.5)	127 (79.9)		66 (75.9)	66 (75.9)	
BMI (kg/m2), n (%)				0.488			0.360
<25	146 (56.8)	53 (54.1)	93 (58.5)		45 (51.7)	51 (58.6)	
≥25	111 (43.2)	45 (45.9)	66 (41.5)		42 (48.3)	36 (41.4)	
Distance from anus, median (IQR), cm	5 (3- 7)	5 (3-7)	5 (3-7)	0.487	5 (3-7))	5 (3-6)	0.401
Tumor differentiation, n (%)				0.250			0.362
Well + moderate	223 (86.8)	82 (83.7)	141 (88.7)		74 (85.1)	78 (89.7)	
Poor	34 (13.2)	16 (16.3)	18 (11.3)		13 (14.9)	9 (10.3)	
Clinical T stage, n (%)				1.000			1.000
T2	6 (2.3)	2 (2.0)	4 (2.5)		2 (2.3)	1 (1.1)	
T3-4	251 (97.7)	96 (98.0)	155 (97.5)		85 (97.7)	86 (98.9)	
Clinical N stage, n (%)				0.060			0.162
N0	21 (8.2)	4 (4.1)	17 (10.7)		4 (4.6)	10 (11.5)	
N+	236 (91.8)	94 (95.9)	142 (89.3)		83 (95.4)	77 (88.5)	
Presurgery CEA (ng/ml), n (%)				0.456			0.163
≤5	190 (73.9)	75 (76.5)	115 (72.3)		69 (79.3)	61 (70.1)	
>5	67 (26.1)	23 (23.5)	44 (27.7)		18 (20.7)	26 (29.9)	
Presurgery CA19-9 (U/ml), n (%)				0.664			0.515
≤37	239 (93.0)	92 (93.9)	147 (92.5)		83 (95.4)	81 (93.1)	
>37	18 (7.0)	6 (6.1)	12 (7.5)		4 (4.6)	6 (6.9)	
Preoperative radiotherapy, n (%)				0.115			0.350
Long-term	239 (93.0)	88 (89.8)	151 (95.0)		80 (92.0)	83 (95.4)	
Short-term	18 (7.0)	10 (10.2)	8 (5.0)		7 (8.0)	4 (4.6)	
nCRT regimens, n (%)				0.490			0.708
Capecitabine	210 (81.7)	78 (79.6)	132 (83.0)		68 (78.2)	70 (80.5)	
Oxaliplatin-containing	47 (18.3)	20 (20.4)	27 (17.0)		19 (21.8)	17 (19.5)	
Adjuvant chemotherapy, n (%)				0.858			0.320
No	77 (30.0)	30 (30.6)	47 (29.6)		29 (33.3)	23 (26.4)	
Yes	180 (70.0)	68 (69.4)	112 (70.4)		58 (66.7)	64 (73.6)	

LNY, lymph node yield; ASA, American Society of Anesthesiologists; BMI, body mass index; CEA, carcinoembryonic antigen; CA19-9, carbohydrate antigen 19-9; nCRT, neoadjuvant chemoradiotherapy.

**Table 2:** Comparison of pathologic outcomes between LNY ≥ 12 group and < 12 group.

Variables	All (n=257)	Before matching			After matching		
		LNY ≥ 12	LNY < 12	*P value*	LNY ≥ 12	LNY < 12	*P value*
		(n=98)	(n=159)		(n=87)	(n=87)
TME quality, n (%)				0.533			0.650
Complete	220 (85.6)	82 (83.7)	138 (86.8)		73 (83.9)	73 (83.9)	
Nearly complete	30 (11.7)	14 (14.3)	16 (10.1)		12 (13.8)	10 (11.5)	
Incomplete	7 (2.7)	2 (2.0)	5 (3.1)		2 (2.3)	4 (4.6)	
No. of LNY, median (IQR)	10 (7-13)	14 (13-16)	7 (6-10)	<0.001	14 (13-16)	7 (6-9)	<0.001
Positive CRMs, n (%)	14 (5.4)	8 (8.2)	6 (3.8)	0.132	7 (8.0)	4 (4.6)	0.350
ypT stage, n (%)				0.163			0.679
T0	45 (17.5)	12 (12.2)	33 (20.8)		10 (11.5)	14 (16.1)	
T1-2	92 (35.8)	40 (40.8)	52 (32.7)		37 (42.5)	35 (40.2)	
T3-4	120 46.7)	46 (46.9)	74 (46.5)		40 (46.0)	38 (43.7)	
ypN stage, n (%)				0.280			0.660
N0	184 (71.6)	67 (68.4)	117 (73.6)		63 (72.4)	59 (67.8)	
N1	55 (21.4)	21 (21.4)	34 (21.4)		18 (20.7)	23 (26.4)	
N2	18 (7.0)	10 (10.2)	8 (5.0)		6 (6.9)	5 (5.7)	
ypTNM stage, n (%)				0.184			0.923
0/PCR	39 (15.2)	10 (10.2)	29 (18.2)		10 (11.5)	10 (11.5)	
I	78 (30.4)	34 (34.7)	44 (27.7)		32 (36.8)	29 (33.3)	
II	68 (26.5)	23 (23.5)	45 (28.3)		21 (24.1)	20 (23.0)	
III	72 (28.0)	31 (31.6)	41 (25.8)		24 (27.6)	28 (32.2)	
TRG, n (%)				0.060			0.677
0	45 (17.5)	12 (12.2)	33 (20.8)		10 (11.5)	14 (16.1)	
1+2	197 (76.7)	77 (78.6)	120 (75.5)		74 (85.1)	70 (80.5)	
3	15 (5.8)	9 (9.2))	6 (3.8)		3 (3.4)	3 (3.4)	
Lymphovascular invasion, n (%)				0.735			1.000
Negative	248 (96.5)	94 (95.9)	154 (96.9)		85 (97.7)	86 (98.9)	
Positive	9 (3.5)	4 (4.1)	5 (3.1)		2 (2.3)	1 (1.1)	
Perineural invasion, n (%)				0.342			0.773
Negative	233 (90.7)	91 (92.9)	142 (89.3)		81 (93.1)	80 (92.0)	
Positive	24 (9.3)	7 (7.1)	17 (10.7)		6 (6.9)	7 (8.0)	
Tumor deposit, n (%)				0.273			0.231
Negative	235 (91.4)	92 (93.9)	143 (89.9)		83 (95.4)	79 (90.8)	
Positive	22 (8.6)	6 (6.1)	16 (10.1)		4 (4.6)	8 (9.2)	

LNY, lymph node yield; TME, total mesorectal excision; CRM, circumferential resection margin; PCR, pathologic complete response; TRG, tumor regression grade.

**Table 3:** Comparison of surgical outcomes between LNY ≥ 12 group and < 12 group.

Variables	All (n=257)	Before matching			After matching		
		LNY ≥ 12	LNY < 12	*P value*	LNY ≥ 12	LNY < 12	*P value*
		(n=98)	(n=159)		(n=87)	(n=87)
Period of operation				0.356			0.165
2010-2015	101 (39.3)	35 (35.7)	66 (41.5)		31 (35.6)	40 (46.0)	
2016-2018	156 (60.7)	63 (64.3)	93 (58.5)		56 (64.4)	47 (54.0)	
Type of surgery				0.421			0.459
LAR	126 (49.0)	48 (49.0)	78 (49.1)		42 (48.3)	36 (41.4)	
ELAPE	108 (42.0)	41 (41.8)	67 (42.1)		38 (43.7)	46 (52.9)	
APR	19 (7.4)	8 (8.2)	11 (6.9)		7 (8.0)	5 (5.7)	
Hartmann’s procedure	4 (1.6)	1 (1.0)	3 (1.9)		0	0	
Surgeons				0.715			0.517
Senior	223 (86.8)	86 (87.8)	137 (86.2)		76 (87.4)	73 (83.9)	
Junior	34 (13.2)	12 (12.2)	22 (13.8)		11 (12.6)	14 (16.1)	
Operative time, median (IQR),min	216 (180-260)	219 (185-261)	215 (180-260)	0.952	215 (180-255)	216 (180-258)	0.680
Blood loss, median (IQR),ml	50 (30-100)	50 (20-100)	50 (30-100)	0.167	50 (20-100)	50 (30-100)	0.092
Conversion to open surgery, n (%)	8 (3.1)	4 (4.1)	4 (2.5)	0.485	3 (3.4)	3 (3.4)	1.000
30-d complications, n (%)	29 (11.3)	9 (9.2)	20 (12.6)	0.403	9 (10.3)	12 (13.8)	0.485
Clavien-Dindo classification, n (%)				0.693			0.744
I-II	23 (8.9)	7 (7.1)	16 (10.1)		7 (8.0)	10 (11.5)	
III-IV	6 (2.3)	2 (2.0)	4 (2.5)		2 (2.3)	2 (2.3)	
Reoperation, n (%)	5 (1.9)	1 (1.0)	4 (2.5)	0.652	1 (1.1)	2 (2.3)	1.000
30-d Mortality, n (%)	1 (0.4)	1 (1.0)	0	0.381	1 (1.1)	0	1.000
Postoperative LOS, median (IQR), d	7 (6-9)	7 (6-9)	7 (6-9)	0.502	7 (6-9)	8 (7-9)	0.180
Follow-up, median (IQR),m	65 (54-75)	67 (52-75)	64 (55-74)	0.778	67 (52-75)	67 (54-72)	0.929
Recurrence*, n (%)	60 (23.3)	23 (23.5)	37 (23.3)	0.971	20 (23.0)	23 (26.4)	0.598
Locoregional recurrence, n (%)	10 (3.9)	5 (5.1)	5 (3.1)	0.512	4 (4.6)	5 (5.7)	1.000
Distant metastasis, n (%)	54 (21.0)	20 (20.4)	34 (21.4)	0.852	17 (19.5)	18 (20.7)	0.85

LNY, lymph node yield; LAR, low anterior resection; ELAPE, extralevator abdominoperineal excision; APR, abdominoperineal resection; LOS, length of stay.

*Four patients had both locoregional recurrence and distant metastasis.

### Comparison of clinical features and survival between different LNY groups

The clinicopathologic characteristics and surgical outcomes were compared between the two LNY groups. The median number of lymph nodes dissected was 14 (13 to 16) in the LNY ≥12 group and 7 (6 to 10) in the LNY <12 group (P<0.001). By comparison, there were no significant differences between the two groups in almost all clinical and pathological features, such as sex, ASA, BMI score, distance from the anus, tumor differentiation, presurgery CEA level, presurgery CA19-9 level, cT, cN, ypT, ypN, TME quality, CRM status and so on (all P>0.05), except that the age of LNY <12 group was older than that of LNY ≥12 group (P<0.001), and LNY <12 group tended to have more TRG 0 cases, while LNY ≥12 group tended to have more TRG 3 cases (P<0.060). There were also no significant differences between the two groups in the administration of nCRT and adjuvant chemotherapy, as well as in surgery-related parameters, for example, the selection of neoadjuvant radiotherapy and chemotherapy regimen, the proportion of patients receiving adjuvant chemotherapy, surgical procedures, type of surgeons, postoperative complications and the like (all P>0.05). After propensity score matching, the clinicopathological characteristics and treatment procedures of the two different LNY groups were almost balanced, so the long-term survival of the two groups was comparable. The results are detailed in **Tables 1**–**3**.

**Figure 1:**
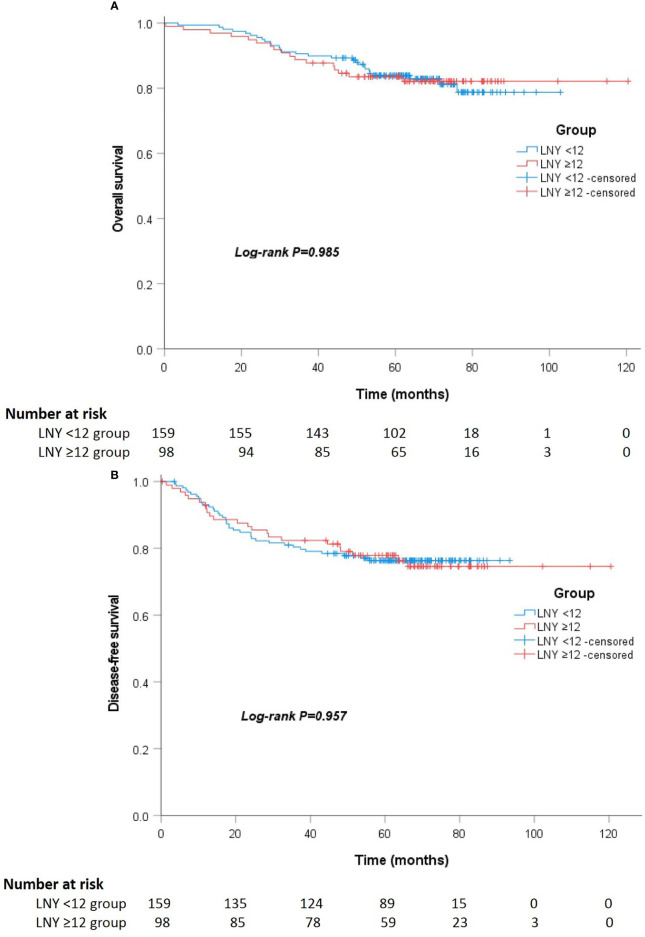
**(A)** Kaplan-Meier curves showing the overall survival stratified by LNY ≥12 and LNY <12 groups in the total cohort (P=0.985). **(B)** Kaplan-Meier curves showing the disease-free survival stratified by LNY ≥12 and LNY <12 groups in the total cohort (P=0.957).

**Figure 2:**
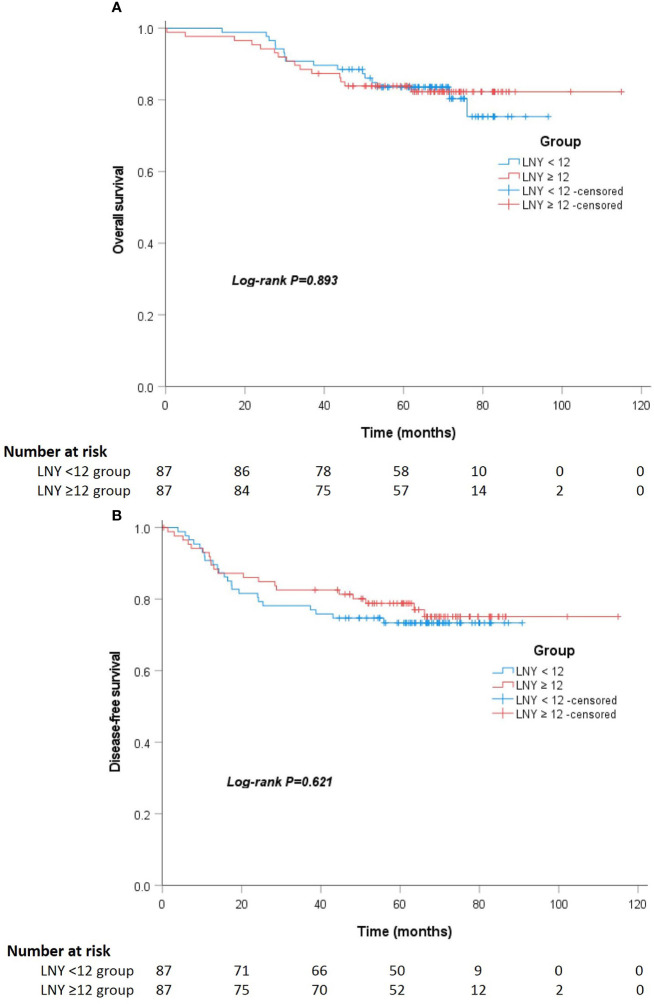
**(A)** Kaplan-Meier curves showing the overall survival stratified by LNY ≥12 and LNY <12 groups in the matching cohort (P=0.893). **(B)** Kaplan-Meier curves showing the disease-free survival stratified by LNY ≥12 and LNY <12 groups in the matching cohort (P=0.621).

After a median follow-up of 67 (52 to 75) and 64 (55 to 74) months, the locoregional recurrence rates of the LNY ≥12 group and LNY <12 group were 5.1% (5 of 98) and 3.1% (5 of 159), and the distant metastasis rates in the two groups were 20.4% (20 of 98) and 21.4% (34 of 159), respectively. There was no significant difference between the two groups (P=0.512 and P=0.852). For all patients, the 3-year OS and DFS was 89.9% and 81.1%, the 5-year OS and DFS was 83.7% and 76.9%, respectively. Separately, the 5-year OS was 83.5% for the LNY ≥12 group and 83.9% for the LNY <12 group (P=0.985, **Figure 1A**), the 5-year DFS was 77.9% and 76.3%, respectively (P=0.957, **Figure 1B**). After further matching, the 5-year OS was 83.9% for the LNY ≥12 group and 83.6% for the LNY <12 group (P=0.893, **Figure 2A**), the 5-year DFS was 78.8% and 73.4%, respectively (P=0.621, **Figure 2B**). Therefore, dissecting fewer than 12 lymph nodes was not significantly related to a poor OS and DFS.

### Factors influencing on lymph nodes dissected and tumor recurrence

The analysis of factors affecting the number of lymph nodes dissected is shown in **Table 4**. Univariate analysis showed that older patient age and long-term radiotherapy were negatively associated with the number of lymph nodes retrieved, while more advanced TRG score and ypN stage were positively associated with the number of lymph nodes dissected (all P<0.05)). On multivariate analysis, only patient age, TRG score and ypN stage independently affected the number of lymph nodes retrieved (P=0.001, P=0.018 and P=0.013). However, there was no significant correlation between the number of lymph nodes dissected and the parameters related to laparoscopic surgery, including the time of operation distribution, surgical procedures, type of surgeons, duration of operation or intraoperative bleeding (all P>0.05). In addition, the TME quality and CRM status were not significantly associated with the number of lymph nodes dissected (all P>0.05).

**Table 4:** Analysis of the association between characteristics of patients and the number of lymph nodes harvested.

Characteristics	B	SE	*P value*
*Univariate analysis*			
Age	-0.102	0.032	**0.001**
Sex (female vs male)	-0.011	0.627	0.987
BMI (≥25 vs<25)	-0.222	0.610	0.716
Distance from anal verge	-0.068	0.130	0.602
Tumor differentiation (poor vs well/moderate)	1.637	0.886	**0.066**
Clinical T stage (T3-4 vs T2)	1.005	2.000	0.616
Clinical N stage (N+ vs N0)	1.847	1.097	**0.093**
Radiotherapy (long vs short-term)	-2.588	1.173	**0.028**
nCRT regimens (oxaliplatin-containing vs capecitabine)	1.072	0.779	0.170
Period of operation (2016-2018 vs 2010-2015)	0.568	0.618	0.358
Type of surgery (no preserving vs sphincter-preserving)	0.058	0.604	0.924
Surgeons (senior vs junior)	-0.010	0.892	0.991
Operative time	-0.001	0.005	0.915
Estimated blood loss	-0.005	0.005	0.357
TRG (3 vs 2, 1 vs 0)	1.849	0.634	**0.004**
ypT stage (T3-4 vs T1-2 vs T0)	0.610	0.403	0.131
ypN stage (N2 vs N1 vs N0)	1.575	0.488	**0.001**
TME quality (Incomplete vs nearly vs complete)	0.617	0.681	0.366
Positive CRMs (yes vs no)	2.009	1.325	0.131
Lymphovascular invasion (yes vs no)	0.479	1.643	0.771
Perineural invasion (yes vs no)	0.020	1.038	0.985
Tumor deposit (yes vs no)	-1.587	1.075	1.141
*Multivariate analysis*			
Age	-0.102	0.031	0.001
TRG (3 vs 2, 1 vs 0)	1.512	0.636	0.018
ypN stage(N2 vs N1 vs N0)	1.227	0.491	0.013

BMI, body mass index; nCRT, neoadjuvant chemoradiotherapy; TRG, tumor regression grade; TME, total mesorectal excision; CRM, circumferential resection margin.

Variables with P values in bold are included in the multivariate analysis.

The analysis of factors influencing tumor recurrence is presented in **Table 5**. Univariate analysis showed that presurgery CEA level, TRG score, ypT stage, ypN stage and tumor deposit were associated with tumor recurrence (all P<0.05). However, on multivariate analysis, only presurgery CEA level and ypN stage were independent predictors of tumor recurrence (P=0.020 and P=0.005).

**Table 5:** Univariate and multivariate analyses of prognostic factors for recurrence.

Variables	Numbers	Univariate		Multivariate		
		Recurrence n (%)	*P value*	HR	95% CI	*P value*
Presurgery CEA (ng/ml), n (%)			0.005			0.020
≤5	190	36 (18.9)		1		
>5	67	24 (35.8)		2.133	1.126-4.042	
TRG, n (%)			0.042			
0	45	5 (11.1)				
1+2	197	49 (24.9)				
3	15	6 (40.0)				
ypT stage, n (%)			0.004			
T0	45	5 (11.1)				
T1-2	92	16 (17.4)				
T3-4	120	39 (32.5)				
ypN stage, n (%)			0.001			0.005
N0	184	32 (17.4)		1		
N1	55	20 (36.4)		2.399	1.210-4.755	0.012
N2	18	8 (44.4)		3.737	1.349-10.351	0.011
Tumor deposit, n (%)			0.010			
Negative	235	50 (21.3)				
Positive	22	10 (45.5)				

CEA, carcinoembryonic antigen; TRG, tumor regression grade; HR, hazard ratio; CI, confidence interval.

## Discussion

Accurate lymph node pathologic assessment is essential to ensure correct staging and treatment of rectal cancer, and it is the strongest predictor of long-term survival (10, 12). Insufficient LNY may result in tumor understaging and suboptimal treatment, ultimately increasing the risk of tumor recurrence (10, 12–14). Moreover, once tumor recurrence occurs, treatment will be extremely difficult (22, 23). nCRT followed by TME surgery is currently considered the standard of care for LARC, however, it has been demonstrated that nCRT may reduce the number of lymph nodes retrieved (7–10). Mechera et al. demonstrated nCRT resulted in a mean reduction of 3.9 lymph nodes and an average reduction in harvested positive lymph nodes of 0.7 compared with patients who received no neoadjuvant therapy (10). This phenomenon may be due to stromal fibrosis and lymph node shrinkage caused by the inflammatory response induced by neoadjuvant radiotherapy, making it difficult to identify lymph nodes in the resected specimen (24, 25). In addition, there are many factors that may affect the number of lymph nodes retrieved, such as the experience and specialization of the surgeon, the experience and work attitude of the pathologist, the characteristics of the patient (age, sex, obesity, etc) and the disease (stage, site, etc) (26).

In our cohort, the median number of lymph nodes retrieved was reduced to 10 (7 to 13) after receiving nCRT, consistent with previous studies (7–9), and approximately 62% of the patients had fewer than 12 lymph nodes retrieved. In order to specifically explore the correlation between the number of lymph nodes dissected and the prognosis of patients after laparoscopic TME, all patients enrolled in this study underwent laparoscopic surgery. Compared with previous reports (16, 17, 27, 28), this group of patients achieved satisfactory results, with a complication rate of 11.3% and mortality of 0.4% within 30 days, the 3-year OS and PFS of 89.9% and 81.1%, and the 5-year OS and PFS of 83.7% and 76.9%, respectively. In recent years, several multicenter studies, such as the COLOR II and COREAN trials, have confirmed that laparoscopic resection of rectal cancer is superior to open surgery in terms of short-term efficacy, and there are no significant differences in oncologic outcomes (16, 17). Data from the COLOR II study showed a complication rate of 40% after laparoscopic surgery, the median number of lymph node dissected was 13 (10 to 18), and the 3-year OS and PFS were 86.7% and 74.8%, respectively (16). In COREAN study, all patients underwent nCRT, the complication rate after laparoscopic surgery was 21.2%, the median number of lymph nodes dissected was 17 (12 to 22), and the 3-year OS and PFS were 91.7% and 72.5%, respectively (17). Clearly, long-term survival was not worse in our cohort than in these two studies, despite relatively fewer lymph nodes being detected.

At present, the impact of the total number of lymph nodes retrieved on the prognosis of rectal cancer patients after nCRT is still controversial. Some studies have suggested that LNY <12 have no effect on the survival of patients receiving nCRT (29, 30), while other studies have come to the opposite conclusion that LNY <12 is a poor prognostic factor for patient survival (31–33). Based on an analysis of 495 rectal cancer patients treated with nCRT, Wang et al. concluded that an LNY of at least 12 indicated an improved survival, so sufficient LNY was still required after nCRT, especially in patients with potentially poor tumor response (29). Lin et al. reported a cohort study of 837 patients underwent nCRT and showed no significant improvement in OS or PFS with ≥12 lymph nodes dissected compared with less than 12 lymph nodes dissected (33). In our study, after propensity score matching, the clinical characteristics and treatment options of the two groups were generally comparable, and there was no significant difference in 5-year OS and PFS between LNY <12 and LNY ≥12 groups. Therefore, the standard of at least 12 lymph nodes being dissected after nCRT remains to be discussed. Some researchers even suggest that since nCRT can reduce tumor size and result in down-grading of lymph node stage, the reduction of LNY is correlated with overall tumor pathologic regression (11, 12). Bustamante-Lopez et al. reported that the number of lymph nodes retrieved was positively associated with laparoscopic surgery and upper rectal cancer, but negatively related to complete or nearly complete pathologic regression, and TRG was the most important factor for decreased LNY (12).

In order to determine which factors might influence the number of lymph nodes dissected after nCRT in patients with rectal cancer, especially those laparoscopic surgery-related parameters, we conducted a multivariable linear regression analysis. The results showed that only uncontrollable factors such as age, TRG score and ypN stage were closely related to the number of lymph node dissected, while laparoscopic surgery-related factors had no effect on this. Here, we took into account the time span of the operation, the qualification of the surgeon, the type of surgery, the quality of TME, the duration of the operation and the like, but none of these controllable factors showed an association with the number of lymph nodes retrieved. The implication may be that in centers where laparoscopic TME can be performed routinely, the number of lymph nodes retrieved after nCRT in rectal cancer is largely determined by patient or disease factors, and laparoscopic surgery itself has little effect. Therefore, it is questionable whether the detection of at least 12 lymph nodes after nCRT can be used as a standard to measure the quality of laparoscopic surgery, and whether it can be considered for cancer-specific prediction. In our study, we further explored the factors affecting tumor recurrence, and the results showed that only presurgery CEA level and ypN stage were independent predictors of tumor relapse, while whether LNY ≥12 or not showed no connection to tumor recurrence.

In view of the fact that LNY <12 may not be a poor prognostic factor for rectal cancer with nCRT, some researchers have proposed that different criteria should be set for this type of patients compared with those without nCRT (33, 34). Lin et al. recommended that at least 7 harvested lymph nodes may be more appropriate for LARC patients with nCRT (33). La Torre el al pointed out that node-negative patients with six or fewer lymph nodes after nCRT were significantly associated with a poor DFS and OS (34). Other researchers have improved pathologic testing reagents or methods to increase the number of LNY, so as to meet the requirements of the current guidelines (35, 36). Dias et al. reported a randomized trial comparing the Carnoy’s solution and formalin concerning LNY in specimens of LARC patients after nCRT, the results showed that the Carnoy’s solution increased lymph node count and reduced the cases with <12 lymph nodes, and it reduced the formalin cases with <12 lymph nodes from 33.8% to 4.6% and upstaged 2 patients (35). In addition, several studies have suggested that other lymph node-related indicators, such as positive lymph node ratio (LNR) or lymph node regression grade (LRG), are more effective in predicting survival of patients with nCRT (37–40). Sun et al. explored the prognostic impact of LRG in LARC patients following nCRT and radical surgery, which ultimately concluded that higher LRG score was associated with higher TRG, more advanced ypT and ypN stages, and poorer OS and DFS, and LRG was an independent prognostic indicator for DFS in LARC patients after nCRT (39).

To our knowledge, this is one of very few studies specifically focusing on whether LNY <12 has adverse impact on the prognosis of LARC patients undergoing laparoscopic surgery after nCRT. In the meantime, the related factors affecting LNY during laparoscopic surgery were analyzed to determine whether surgery-related parameters would have influence on the number of lymph nodes harvested. Therefore, this study could provide more data for these controversial issues. However, the present study has several limitations. First, due to the retrospective design and small sample size, there must be inherent selection bias, and the generality of the conclusion is uncertain. Second, the time span of this study was 9 years, and the development of new radiotherapy techniques, chemotherapy regimens and laparoscopic techniques may have an impact on the prognosis of patients. However, the deviation caused by these factors was offset to some extent by multivariate analysis. Finally, more than 60% of the patients in this cohort had less than 12 lymph nodes retrieved, which may be related to the sampling method and work attitude of pathologists, and this could be changed by some methods.

## Conclusion

Our study identified that for LARC patients who underwent laparoscopic surgery after nCRT, the number of lymph nodes dissected less than 12 has not been proved to be an adverse predictor for long-term survival. There were a number of factors associated with LNY after laparoscopic TME, but mainly patient and disease related factors, such as age, TRG score and ypN stage, while laparoscopic surgery-related factors had no effect on this. Given the relatively small sample size of this study, more convincing conclusions need to be confirmed by more large-scale clinical studies.

## Data availability statement

The datasets presented in this article are not readily available because: Due to the sensitive nature of the clinical data collected in this study, patients were assured raw data would remain confidential and would not be shared, except under specific request. Requests to access the datasets should be directed to suxiangqian@bjmu.edu.cn.

## Ethics statement

The studies involving human participants were reviewed and approved by The Research Ethics Committee of Peking University Cancer Hospital & Institute. The patients/participants provided their written informed consent to participate in this study. Written informed consent was obtained from the individual(s) for the publication of any potentially identifiable images or data included in this article.

## Author contributions

Study conception and design: HY and XS. Acquisition of data: HY, CZ, ZY, and XW. Analysis and interpretation of data: HY, JX, MC, and BJ. Writing manuscript: HY. All authors contributed to the article and approved the submitted version.
